# Serum Zinc Levels in Iron Deficient Women: A Case-Control Study

**DOI:** 10.4274/tjh.2015.0206

**Published:** 2016-05-16

**Authors:** Onur Özhan, Neslihan Erdem, İsmet Aydoğdu, Ali Erkurt, İrfan Kuku

**Affiliations:** 1 Çukurova Dr. Aşkım Tüfekçi State Hospital, Clinic of Endocrinology, Adana, Turkey; 2 Celal Bayar University Faculty of Medicine, Department of Internal Medicine, Manisa, Turkey; 3 Celal Bayar University Faculty of Medicine, Department of Hematology, Manisa, Turkey; 4 İnönü University Faculty of Medicine, Department of Hematology, Malatya, Turkey

**Keywords:** iron, Zinc, Women, Iron deficiency, Anemia

## Abstract

Since similar symptoms and findings can be seen in the deficiencies of both iron and zinc, we aimed to evaluate the serum zinc levels of women with iron deficiency anemia (IDA). This study was conducted with women with iron deficiency and a healthy control group. When serum zinc levels were compared, they were found to be lower in the IDA group, which was statistically significant. With the help of these studies, iron and zinc treatment instead of only iron replacement may be considered in cases of iron deficiency.

## Abstract (tr)

Demir ve çinko eksikliği benzer belirti ve bulgularla giden hastalıklar olması sebebiyle, demir eksikliği anemisi (DEA) olan kadınlarda serum çinko düzeylerinin değerlendirilmesini planladık. Bu çalışma DEA’lı kadınlar ile sağlıklı kontrol olarak alınan kadınlar üzerinde yapıldı. Serum çinko düzeyleri karşılaştırıldığında, DEA grubunda istatistiksel olarak düşük saptandı. Bu çalışmaların yardımıyla sadece demir değil, demir ve çinkonun beraber tedavisi demir eksikliği olan olgularda düşünülebilir.

## INTRODUCTION

Iron deficiency anemia (IDA) is the most common anemia around the world and a public health concern in developing countries that still suffer from malnutrition problems [1,2]. Similar findings and symptoms affecting various systems in the body may be found both in iron and zinc deficiency; moreover, coexistence of these deficiencies can exaggerate the symptoms. However, there are not enough studies about zinc levels in adult anemic subjects. Therefore, we aimed to evaluate the serum zinc levels of women with IDA and investigate whether serum zinc levels in women with iron deficiency were low or not.

## MATERIALS AND METHODS

Thirty women between 18 and 60 years of age with iron deficiency who had presented to our outpatient clinics were enrolled as the patient group and a healthy group consisting of 30 women with the same age range served as the control group. The study was conducted in the İnönü University Faculty of Medicine, Department of Internal Medicine. Women with history of infection within 1 month, or with chronic diseases were excluded.

Diagnosis criteria for iron deficiency were hemoglobin below 12 g/dL and serum ferritin level below 20 µg/dL [3]. The normal values of serum zinc levels were between 70 and 120 µg/dL [4]. Iron and iron-binding capacity were measured by the calorimetric method with an Olympus OSR6186 (Germany), whereas serum ferritin levels were measured by nephelometric method with a 33 Dade Behring (Germany). Complete blood count analysis was performed with a Beckman Coulter LH 750 analyzer (USA). Serum zinc levels were measured by atomic absorption method with the PerkinElmer Analyst 800 (Germany).

### Statistics

Results were given as ± standard deviation, and with 95% safety and distribution. Statistical analysis was conducted with SPSS and the independent sample t-test.

## RESULTS

Thirty healthy women and 30 women with iron deficiency were included in this study. Ages of the women were between 18 and 60; mean age was 38.4±10.5 years in the IDA group and 39.8±12.5 years in the control group. There was no statistical difference between the groups (p>0.05).

Mean hemoglobin level was 10.1±1.4 g/dL in the IDA group and 14.1±0.5 g/dL in the control group. There was a statistically significant difference between the groups (p<0.001). Hematologic parameters of each group are given in Table 1.

Serum iron, ferritin, and transferrin saturation levels, which are the parameters helping in the diagnosis of IDA, were higher in the control group compared to the IDA group. There was a statistically significant difference between the groups (p<0.001). Iron levels and iron storage parameters are given in Table 2.

When the control and IDA groups were compared, serum zinc levels were found to be decreased as serum iron levels decreased. Minimum serum zinc level was 34 µg/dL while the maximum was 84 µg/dL in the IDA group, and mean serum zinc level was 55.8±10.8.

In the IDA group, serum zinc level was in the normal range (70-120 µg/dL) in only three of the patients. The remaining 27 patients’ serum zinc levels were below 70 µg/dL. On the other hand, in the control group, only one individual had a serum zinc level below 70 µg/dL, while the remaining subjects’ serum zinc levels were in the normal range. There was a statistically significant difference between the groups (p<0.0001). Serum zinc levels of the IDA and control groups are compared in Table 2.

## DISCUSSION

Although trace elements are found in minimal quantities, they have important roles in homeostasis. Two of the most important trace elements are iron and zinc. IDA is still a serious problem in Turkey and around the world [1,5,6]. Considering many etiological factors like low socioeconomic status, malnutrition, high-fiber diet, pica disorder, parasitic infections, and milk allergies, it is not a surprise to see zinc deficiency and iron deficiency at the same time [2,7,8].

Coexistence of iron and zinc deficiency has attracted the interest of researchers and there have been many studies about this subject. Furthermore, this situation had led to the following question: Are there any interactions between these two elements?

One of the reasons for iron deficiency occurring with zinc deficiency, other than diet, is the increase in production of Zn-protoporphyrin and usage of zinc instead of iron in the protoporphyrin structure [9].

There are also other hypotheses that zinc deficiency can cause iron deficiency. It has been shown that, in animal studies with mice and rats with zinc deficiency, bone marrow erythrocyte progenitors and plasma erythropoietin levels are decreased [10,11,12]. Furthermore, there are also hypotheses that zinc deficiency can make the erythrocytes vulnerable to oxidative stress, which can cause anemia [13,14].

In another study, serum zinc levels were measured in children between 1 and 14 years of age with iron deficiency. The serum zinc levels were lower in the IDA group than the control group (p=0.017). There was a statistically significant difference between the two groups, as in our study. As a result, it has been suggested that serum zinc levels should be checked in children with iron deficiency [15].

Serum zinc levels were studied in children with iron deficiency in Ankara. When zinc deficiency was accepted as levels below 2 SDs of mean levels of the control group, among the 100 anemic patients 23 patients had zinc levels of less than 1 SD and 19 patients had zinc levels of less than 2 SDs of the mean levels. These results were compatible with our results. It was also suggested that zinc deficiency should be evaluated in patients with IDA, because of the similarity of the symptoms of these two deficiencies [7].

In Arcasoy’s study in 1985, histopathological changes causing iron and zinc deficiency in intestinal mucosa were reversed with zinc treatment and the absorption of zinc and iron were improved [2].

Because of the lack of studies regarding this subject in adult women and the high prevalence of IDA in this age group, we conducted a study with women between 18 and 60 years of age. We tried to find out whether zinc deficiency coexists with iron deficiency or not. Although the studies we have mentioned here mostly involved children, our study has shown similar results.

A study conducted in Vietnam showed that underprivileged women were at increased risk of insufficient micronutrient intake due to poor diet quality [16]. The effects of supplementation in children have also been studied. It was found that supplementation with iron plus zinc improved serum zinc and plasma ferritin [17].

Similar findings and symptoms affecting various systems in the body may be found both in iron and zinc deficiencies; therefore, the levels of zinc must be carefully evaluated in cases of iron deficiency. More importantly, as iron deficiency is still an existing problem in Turkey, further studies investigating the interactions between these elements must be performed. We suggest that serum zinc levels should be evaluated in adult women with IDA, but further studies are needed to evaluate the benefit of simultaneous zinc and iron treatment instead of only iron treatment in this age group.

## Ethic

This study is ethically approved by İnönü University’s Local Ethics Committee.

## Figures and Tables

**Table 1:**
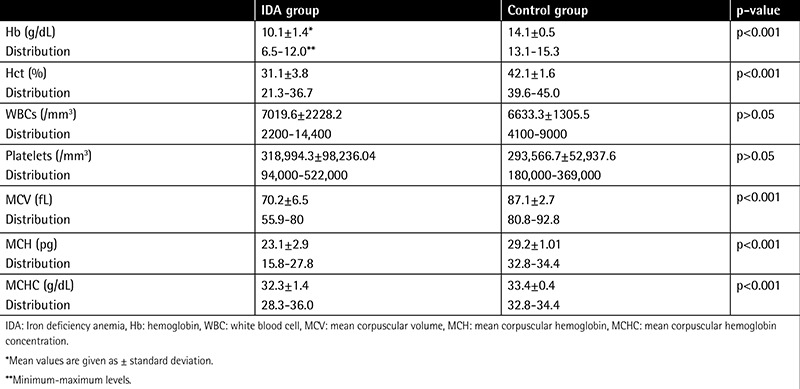
Hematologic parameters of the iron deficiency anemia and control groups.

**Table 2:**
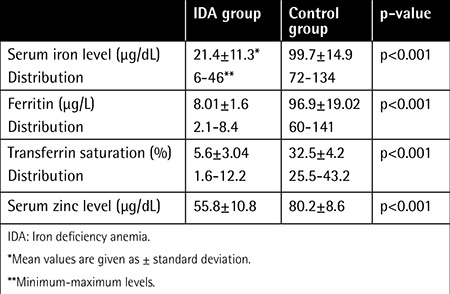
Iron levels, zinc levels, and iron storage parameters of the groups.
